# A potential new treatment with upadacitinib for acquired reactive perforating collagenosis

**DOI:** 10.1016/j.jdcr.2024.04.017

**Published:** 2024-04-20

**Authors:** Wei Ding, Yuting Wang, Linyi Song, Naihui Zhou

**Affiliations:** Department of Dermatology, the First Affiliated Hospital of Soochow University, Suzhou, Jiangsu Province, China

**Keywords:** acquired reactive perforating collagenosis, dermatology, Janus kinase inhibitors, pruritus, treatment, upadacitinib

## Introduction

Acquired reactive perforating collagenosis (ARPC), a form of perforating dermatosis, has an unknown etiology and pathogenesis and is frequently associated with systemic disorders. It often manifests as umbilicated hyperkeratotic papules or as a dome-shaped lesion with a central crater. Diagnosis is based on clinical presentation and characteristic histopathologic findings. Treatments including topical steroids, oral antihistamines, allopurinol, UV-B phototherapy, and dupilumab have been reported as effective but can be unpredictable.^1^

Janus kinase inhibitors are novel small molecules developed for treating various autoimmune and inflammatory diseases. Upadacitinib is an oral selective Janus kinase 1 inhibitor, which has emerged as a Food and Drug Administration–approved therapeutic option for atopic dermatitis^2^ and is used as an off-label treatment for chronic prurigo.^3^ A case study is presented of a 63-year-old man with ARPC characterized by extensive involvement and intense pruritus that responded well to treatment with upadacitinib.

## Case report

**Fig 1:**
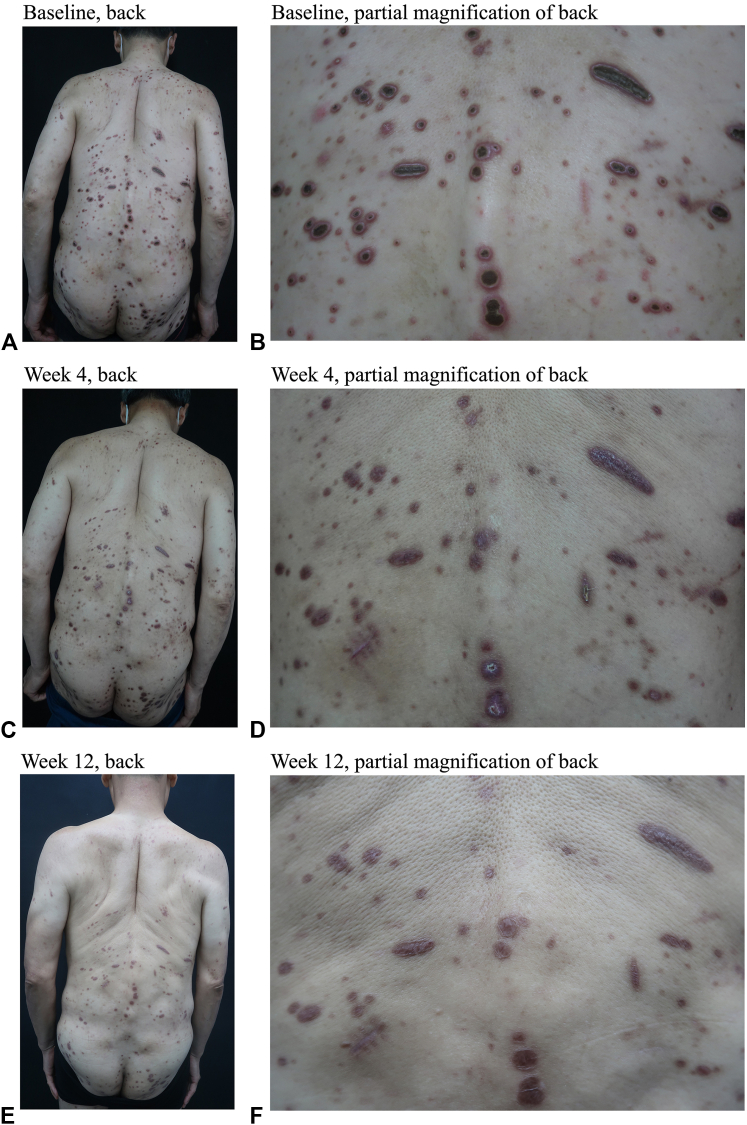
Back of patient at (**A, B**) baseline, (**C, D**) week 4, and (**E, F**) week 12.

A 63-year-old man presented to the clinic with a 3-month history of generalized umbilicated hyperkeratotic lesions over the trunk and extremities, seen in Fig 1. His pruritus dramatically affected his daily activities and sleep. Topical steroids, antihistamines, thalidomide, and phototherapy had been tried but were ineffective. Systemic steroids were not advised due to his history of poorly controlled type 2 diabetes mellitus.

On physical examination, multiple hyperkeratotic red or brown papules and plaques were noted on the trunk and extremities primarily on extensor surfaces, with central umbilicated necrosis, accompanied by Koebner phenomenon. His laboratory reports were unremarkable except for hyperglycemia.

**Fig 2:**
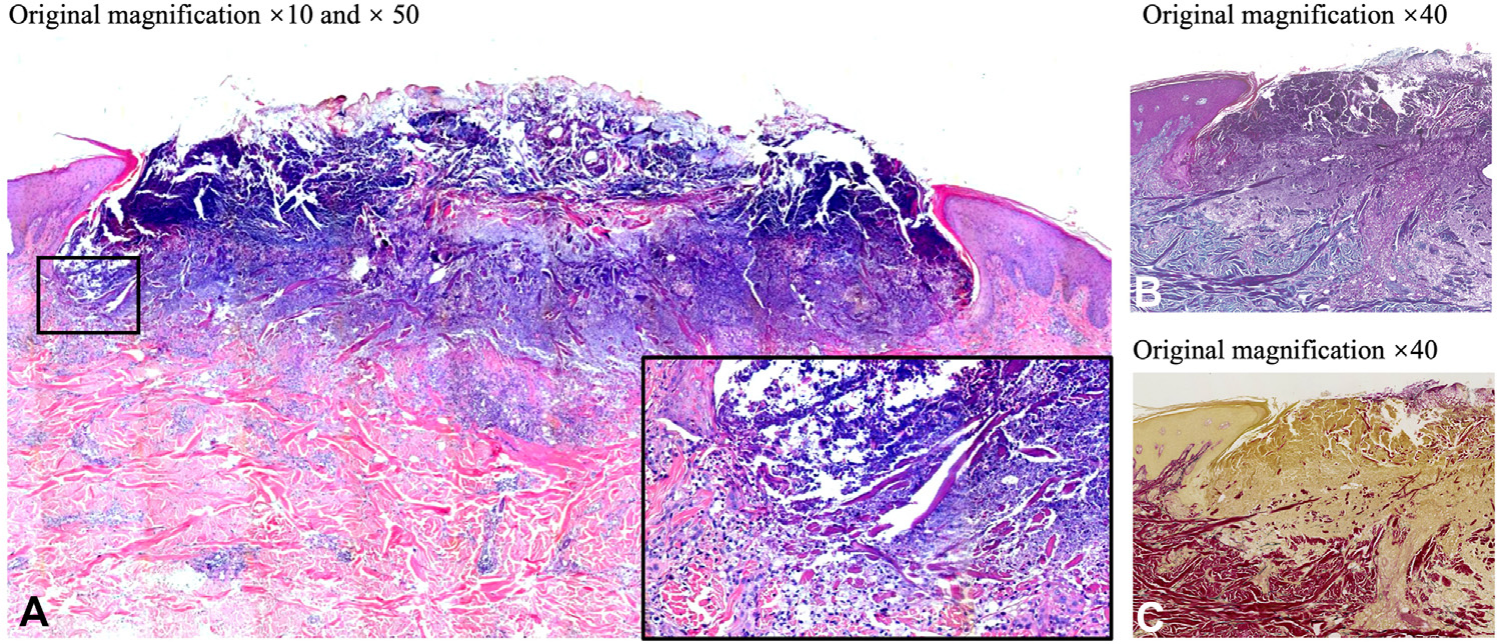
Histopathology. **A,** A cup-shaped epidermal depression with a central crater contains degenerated collagen bundles, inflammatory cells, and cellular debris, and the surrounding epidermal shows acanthosis. Vertically orientated collagenous fibers penetrate the epidermis. **B, C,** Masson trichrome staining and Verhoeff-van Gieson staining showed collagen fibers but no elastic fibers penetrated the epidermis. (**A** and **inset of A,** Hematoxylin-eosin stain; **B,** Masson trichrome stain; **C,** Verhoeff-van Gieson stain; original magnification: **A,** ×10; **inset of A,** ×50; **B** and **C,** ×40.)

Histopathology showed typical collagen bundles perforating through the epidermis. Masson trichrome staining was positive and Verhoeff-van Gieson staining was negative. It showed that no elastic fibers but collagen fibers penetrated the epidermis, as seen in Fig 2, and a diagnosis of ARPC was made. Given the severe involvement, intense pruritus, and his refusal of dupilumab as a therapeutic option, upadacitinib was started at 15 mg once daily after the screening tests.

Substantial improvement in the pruritus was reported 1 week after treatment where almost all skin lesions had flattened and residual post inflammatory hyperpigmentation was presented with some lesions leaving scars at the 12 week follow-up. A slightly elevated liver function was reported, but it returned to normal after 2 weeks. His pruritus intensity on a 0 to 10 numerical rating scale was 10 at baseline, 5 after the first week, and 0 at the fourth week follow-up. Upadacitinib was reduced to 15 mg every other day after 12 weeks for maintenance therapy.

## Discussion

Given that ARPC presents a therapeutic challenge, it is worth exploring the potential pathogenesis and therapy. Pruritus plays a central role in the etiology of ARPC, although its mechanism remains to be fully elucidated.^4^ In the case presented, the patient’s pruritus probably resulted from sensory neuropathy due to his underlying diabetes, as it is the most commonly associated condition of ARPC, followed by renal failure and hepatic insufficiency.^5^ The microtrauma due to subsequent scratching also triggers a severe itch-scratch cycle, exacerbating the condition.^4^ Successful antipruritic treatment,^6^ along with optimal management of any associated internal or oncologic disease is reported as an important requirement for the successful treatment of ARPC.^7^

In a retrospective cohort study, predominance of T helper 2 cells and the upregulated cytokines IL-4 and IL-13 were observed in ARPC, indicating the involvement of the T helper 2 axis immune response in its pathogenesis.^1^ Given that the Janus kinase/signal transducers and activator of transcription signal mediates T helper 2 cytokines, regulates the epidermal barrier, and modulates peripheral nerves of pruritus transduction,^8^ upadacitinib should be a promising potential therapy for refractory ARPC, despite the limitation of a single case report.

## Conclusion

This real-life experience of upadacitinib treatment in a patient with severe ARPC illustrates an excellent response on relieving pruritus, resolving skin lesions, and improving quality of life. Although further evaluations with larger studies are needed, upadacitinib could represent a novel and rapid alternative treatment for severe ARPC.

## Conflicts of interest

None disclosed.
